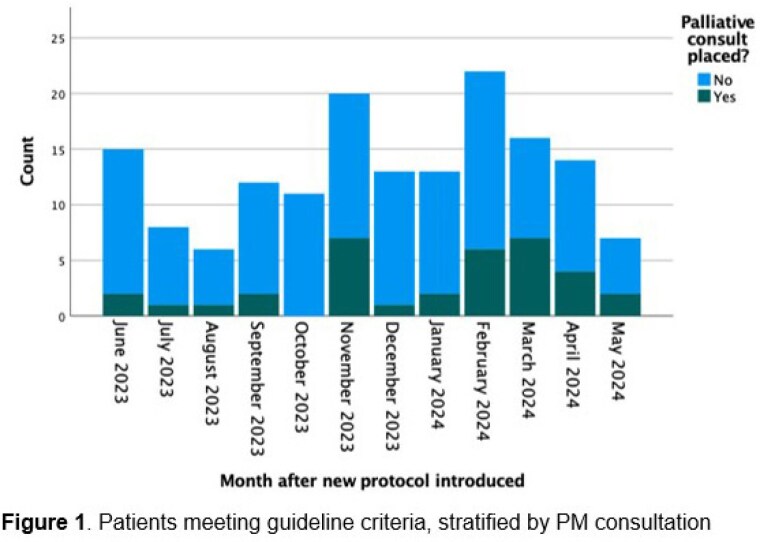# 77 Implementation of a Palliative Consultation Process at an Urban Burn Center: Lessons & Optimizations

**DOI:** 10.1093/jbcr/iraf019.077

**Published:** 2025-04-01

**Authors:** Carey Lamphier, Lauren Nosanov, Yuk Ming Liu, Jasmin Mercedes, Laura Johnson

**Affiliations:** Grady Hospital; Emory University School of Medicine; Emory University - Walter L. Ingram Burn Center; Grady Hospital; Walter L. Ingram Burn Center at Grady Memorial Hospital

## Abstract

**Introduction:**

Burn injuries are life altering events for both patients and families. Early Palliative Medicine (PM) involvement can assist with goals of care planning, symptom management, and emotional and spiritual support, augmenting the holistic multidisciplinary care provided by the Burn team. Identification of need was identified as a quality improvement opportunity given historically low rates of PM consultation.

**Methods:**

Implementation of a PM consultation guideline occurred at a high volume, urban Burn referral center, using criteria previously suggested in the literature. All patients admitted to the Burn service after June 2023 were included in a prospectively collected registry which included patient demographics, injury specific characteristics, and clinical outcomes. Injury mechanisms were characterized as burn or non-burn and guideline criteria met for each patient were tracked. Descriptive statistics were reported, the burn and non-burn groups were compared, and binary logistic regression was performed to identify independent predictors of PM consultation in the burn-injured cohort.

**Results:**

546 patients were identified in the one-year study period, with 523 available for analysis after exclusion of readmissions. Patient age ranged from 3 months to 94 years (median (IQR) 45 (32) years) and presented a median of 0 days post-injury (IQR 1 day). For burn-injured patients, the Total Body Surface Area (TBSA) range was 0-95% (median (IQR) 5.5% (12.0%)), with median modified Baux score of 57.0 (IQR 38.5). The non-burn group consisted primarily of rash and skin infections (61.6%) and friction and shear injuries (25.3%). Both cohorts were demographically similar apart from a higher proportion of males in the burn group (p = 0.026). While 14 PM consults were placed in the six months pre-implementation, 42 consults in total were placed during the study period, representing a 1.5-fold increase. While 148 patients met criteria for consultation, 33 (22.3%) had PM consultations placed (19.8% of eligible burns, 31.3% non-burns). The most commonly met criteria were age > 70 years and high burden of comorbidities, however, on regression analysis only a modified Baux score >110 independently predicted PM consultation (AOR 12.8, CI 3.6-46.1, p< 0.001).

**Conclusions:**

Criteria for PM consultation adapted from the implementation literature resulted in increased utilization of Palliative Care for our high-volume Burn Service, yet there remain a number of patients who would benefit from consultation who do not receive them due to relatively low guideline compliance. Future steps in our quality improvement efforts will target identification of contributing factors. Next steps include initiation of an Electronic Medical Record “Best Practice Alert” for the clinical team following nursing-driven screening of patient eligibility.

**Applicability of Research to Practice:**

Standardization of PM consultation should improve access to this resource for all patients.

**Funding for the Study:**

N/A